# Rational and Irrational Issues in Breast Cancer Screening

**DOI:** 10.3390/cancers3010252

**Published:** 2011-01-11

**Authors:** Cornelia J. Baines

**Affiliations:** Dalla Lana School of Public Health, University of Toronto, 155 College Street, Ste 576, Toronto, Ontario, M5T 3M7, Canada; E-Mail: cornelia.baines@utoronto.ca; Tel.: 416-978-1458; Fax: 416-978-2087

**Keywords:** breast screening, screening mammography, randomized controlled trials

## Abstract

Evidence on the efficacy of breast screening from randomized controlled trials conducted in the last decades of the 1900s is reviewed. For decades, controversy about their results has centered on the magnitude of benefit in terms of breast cancer mortality reduction that can be achieved. However more recently, several expert bodies have estimated the benefits to be smaller than initially expected and concerns have been raised about screening consequences such as over-diagnosis and unnecessary treatment. Trials with substantial mortality reduction have been lauded and others with null effects have been critiqued. Critiques of the Canadian National Breast Screening Study are refuted. Extreme responses by screening advocates to the United States Preventive Services Task Force 2009 guidelines are described. The role vested interests play in determining health policy is clearly revealed in the response to the guidelines and should be more generally known. A general reluctance to explore unexpected results or to accept new paradigms is briefly discussed.

## 1. Introduction

The idea that early detection of breast cancer must be beneficial is totally compelling. Ideally, screening with mammography will achieve earlier detection of breast cancer. “Earlier detection” implies earlier than would normally be achieved in routine clinical circumstances. The assumptions are that earlier detection will alter the natural history of the disease, namely that death from breast cancer will be prevented and that there will be no major adverse effects from screening itself.

What are some underlying issues hidden in the above paragraph?

Screening mammography is not the same as diagnostic mammography. The former screens normal women for early-stage cancer and is directed at specific age groups. Diagnostic mammography evaluates abnormalities in symptomatic women of any age.

The early detection achievable with mammography is not early enough to alter the natural history of breast cancer in all women with breast cancer. Mammography trials reveal at best a 30% reduction in breast cancer mortality. This means that for every 100 women destined to die of breast cancer if not screened, 70 will still die even if they are screened.

Screening unavoidably increases the incidence of breast cancer. This increase is not due to radiation exposure from mammography but due to lesions being identified as breast cancer that otherwise would never have been diagnosed, namely over-diagnosis.

Most important is that the majority of women who are diagnosed with breast cancer will not die of breast cancer.

## 2. The Advantages of Randomized Controlled Trials

To establish the benefit of screening, the single most powerful tool is the randomized controlled trial. It is not sufficient to establish that early detection is associated with longer survival time post-diagnosis. One wants to demonstrate that the early detection achieved by screening is associated with a lower risk of dying of breast cancer compared to what happens in women who are not screened. To achieve this ‘gold standard’ of evidence, a number of randomized screening trials were launched in the last four decades of the 20th century in Europe, the U.S. and Canada.

Some important elements of the randomized controlled trial are:

The design of the trial should be ethically approved.

Participants in a trial should all sign informed consent.

Randomization should achieve comparability across the two groups that are to be compared. This means that important variables—both known and unknown—are likely to be equally distributed. In the case of screening trials, women receiving one intervention (in this case mammography) should be similar (with respect to age, marital status, age at menarche, age at first birth, *etc.*) to those randomized as controls (no mammography).

There should be quality control in terms of the intervention tested.

Participant compliance with the intervention should be reported.

Outcome assessment should be blindly assessed. For screening trials, the outcome of interest was death due to breast cancer.

In spite of these principles, methodological weaknesses occurred in all trials and some will be discussed later. But first it is useful to briefly review the trials that were conducted.

## 3. A Summary of the Screening Trials

Screening mammography was first evaluated in the *New York Health Insurance Plan Study (HIP)*. In 1963, it randomized (without informed consent) women aged 40 to 64 with about 30,000 receiving annual two-view mammography and clinical breast examination for three screens and another 30,000 serving as controls who would receive ‘usual care’. Thirty-five percent of the study group did not attend first screening but were included in the intention-to-treat analysis. Although mammography at that time did not match current standards, the HIP study's 15 year follow-up revealed an overall statistically significant reduction in breast cancer mortality of 23% but no benefit was seen from screening women age 40–49 [1]. An unwelcome transient and paradoxical increase in breast cancer mortality was observed in women 40–49 who received screening compared to no screening although it was not statistically significant [2]. Because this paradox would also be observed in subsequent screening studies it deserves attention.

*The Edinburgh (UK) trial* was initiated in 1976, recruiting women aged 45–64 years with 23,226 in the study group and 21,904 in the control group [3]. The process of randomizing general practice units was never described but unfortunately it resulted in a serious discrepancy in socio-economic status: it was much higher in the screening practices than in the controls. The inevitable consequence was that all-cause mortality was much higher in the control group; with successful randomization it would have been equal. For this reason, the study cannot contribute to an understanding of the efficacy of screening.

In 1976, another study was initiated in *Malmö, Sweden*, for those aged 45–69 years, with about 21,000 women in the study group and another 21,000 in the control group. After 12 years, a statistically non-significant breast cancer mortality reduction of 19% was observed [4].

*The Swedish Two-County trial* had two components.

The Őstergötland trial began in 1977 involving women aged 40–74, with approximately 39,000 in the study group and 37,500 in the control group. (As with the HIP study, the Őstergötland trialists reported variable numbers of participants in sequential publications.) Randomization was by geographic cluster rather than by individual. The intervention was single-view mammography every two years for women less than 50 years of age and every 33 months for women 50 and over. At 12 years follow-up, the breast cancer mortality reduction encompassing all ages was 18%, again statistically not significant [5].

The Kopparberg trial also began in 1977, recruited women aged 40 to 74 and used geographic clustering for randomization. The sample size reported varied over publications, but approximately 38,800 women were in the study group and 18,600 were in the control group. The intervention was the same as for the Őstergötland trial. At 12-year follow-up, a statistically significant reduction in mortality of 32% was observed for the entire study population. At 14-year follow-up, there was still no significant breast cancer mortality reduction in women aged 40-49 in either county [6].

Up until this point, with the exception of the HIP study, screening trials compared mammography screening to no screening. This was about to change. By 1979, those interested in screening in North America had concluded that the HIP study had lacked the power to demonstrate a screening benefit in women age 40–49 because breast cancer incidence is so much lower in younger than in older women. Clearly, more women in the 40–49 year age group were required to participate in a trial. The HIP study also raised the question: what was the incremental benefit of mammography over and above clinical examination of the breasts (CBE)? Concurrently in the U.S., there had been a huge project, the Breast Cancer Demonstration Project; it delivered screening but it was not a randomized controlled trial. The U.S. National Institutes of Health and the American Cancer Society convened a working group to evaluate available data on breast cancer screening [7]. Two recommendations emerged. The first was that a larger study was needed to determine whether screening was effective in women aged 40–49: the screening intervention should be combined mammography and CBE and with the comparison being screening *versus* no screening. The second recommendation was that the incremental effect of mammography over and above CBE (in reducing breast cancer mortality) needed to be studied in women aged 50–59. This required comparison of women receiving combined mammography and CBE with women receiving CBE alone.

These two recommendations determined the design of the *Canadian National Breast Screening Study (CNBSS)* which commenced in 1980. By 1980, informed consent had become an essential component of human trials in Canada. The need for informed consent, combined with the research questions intrinsic to the recommendations, led to much criticism both at the inception of the two trials and later when results were published. So intense was the criticism in 1980–1981 for exposing women to mammography radiation that it seriously hampered recruitment and threatened the continuation of the study [8]. As a consequence of these delays, instead of receiving five annual screens, women who entered late in the recruitment phase received only four.

Beginning in 1980, CNBSS-1 recruited women aged 40 to 49 years with 25,214 in the study group and 25,216 in the control group. The numbers did not vary over publications. The intervention was four or five annual two-view mammograms with CBE. Controls received one CBE on entry (a consequence of the requirement for informed consent) and thereafter controls responded to annual questionnaires and had usual care in the community. Individual randomization (methodologically preferable to cluster randomization) occurred after CBE was performed by a nurse-practitioner. The randomization process was the responsibility of the center co-ordinators who were blind to the CBE results. At 13-year follow-up, mortality reduction was a statistically non-significant 3% [9].

CNBSS-2 also began in 1980 recruiting women age 50 to 59 years with 19,711 in the study group and 19,694 in the control group. The study group received four or five annual two-view mammograms and CBEs while the controls received four or five annual CBEs. The relative risk of breast cancer death comparing screened women to control was 1.02 – clearly no benefit was gained from mammography screening [10]. The protocol included instruction in breast self examination (BSE) for both age groups. The results of BSE instruction in both groups have been published [11,12].

CNBSS-1 and -2 were conducted in 15 screening centers located across Canada from Halifax to Vancouver. All were based in teaching hospitals or in cancer centers where the best expertise in radiology and surgery was available. The central administration unit was based at the University of Toronto. Surgeons and radiologists met annually in Toronto. Both the director (Anthony B. Miller) and the deputy director (Cornelia J. Baines) visited the centers frequently to observe operations and enhance performance when necessary.

In the meantime, other screening trials based on a comparison of screening *versus* no screening were being conducted in Europe. In 1981, the *Stockholm, Sweden trial* started recruiting women aged 40 to 64 years. Cluster randomization by birth date was employed and the numbers in the study and control groups changed between published reports. The intervention was single-view mammography done twice with an interval of 28 months. No mortality reduction was reported in women aged 40–49 after 11.4 years follow-up [13].

In 1982, the *Göteborg, Sweden trial* started recruiting women aged 39 to 59 years, with 21,650 in the study group and 29,961 in the control group. A complex form of cluster randomization was employed and interpretation is complicated. At 12-to 14-year follow-up, no statistically significant reduction in breast cancer mortality was observed and, indeed, for women aged 50–54 no reduction was observed at all according to (www.cancer.gov/cancertopics/pdq/screening/breast/HealthProfessional/page5). In contrast, others have reported a 48% significant reduction for women aged 45–49 [14].

In 1986, the Forrest Report [15] recommended the introduction of a *National Health Service Breast Screening Program* in the United Kingdom offering mammography once every three years to women aged 50 to 64. In 1988, this breast screening program was set up in England and more than 110,700 women between the ages of 50 and 64 were invited for screening. However, programs are not trials: they offer services to targeted women in the population.

The most recently initiated trial, the *UK Age trial*, operated between 1991 and 1997 and was designed to avoid the “age creep” that had affected all the other trials whereby women recruited in their late 40s progressed into their 50s soon after recruitment [16]. However, trial analysis was always based on age at entry, not age at diagnosis. The concerns were that because most women under 50 were premenopausal and those over 50 post menopausal, this biological difference might influence screening outcomes. By recruiting 160,291 women aged 39–41 and randomizing them in a ratio of 1:2 to screening *versus* a control group, the U.K. researchers recruited a study population that remained in their 40s at 10-year follow-up. Although a reduction of 17% was observed in breast cancer mortality at a mean follow-up of 10.7 years, it was not statistically significant and the absolute risk reduction was minute. Unfortunately the researchers have not revealed annual cumulative breast cancer mortality as have other trialists, so we do not know if the mortality paradox occurred in this trial as in others [2].

## 4. What Explains the Lack of Consistency in Trial Results?

Compliance in attendance at first screen varied from 61% to 100% across trials.

In some trials single-view mammography was used; in others two-view.

In some trials clinical examination of the breast was included, in others not.

The frequency of screening varied from 12 to 33 months

The total number of screens performed varied from two to ten.

Ages of entry ranged from 39 up, depending on the study.

Randomization was sometimes by cluster (geographic region, birth date, or medical practice) and sometimes by individual, the latter being the gold standard.

Only two trials showed substantial breast cancer mortality reductions; the HIP Study and the Swedish Two-Country trial. There are several possible explanations. If the stage at detection is advanced in controls, as occurred in these two studies, the potential for screening benefit is enhanced. In contrast, in the CNBSS, with controls having less advanced disease at diagnosis than in the other two studies, there was less potential for benefit to be shown from screening. Furthermore, benefit from screening was likely to be less in Canada in the 1980s because all women with axillary node-positive disease were routinely offered adjuvant hormone and chemo-therapy [17] while in Sweden this was not the case.

The mode of outcome analysis is also an important factor. If deaths from breast cancer are determined by an external expert panel, as what happened in the CNBSS and the HIP study, outcome validity is likely to be more valid.

After many years of follow-up, the trials overall demonstrate about a 15 to 16% reduction in breast cancer mortality [18,19]. Looking specifically at the benefit from screening women aged 40–49, the overview of Swedish trials [20] revealed a 9% reduction which was not statistically significant, the U.K. trial a 17% reduction, again not significant [16], and the United States Preventive Services Task Force (USPSTF) [18] a 15% reduction again statistically not significant. Translating that proportionate reduction into the absolute benefit, there is about one breast cancer death prevented per 2,000 women screened for 10 years. When that benefit has to be balanced against the 25% of screen-detected cancers that are over-diagnosed (discussed later) [21] and against inevitable and unnecessary treatment, the benefits of screening are somewhat muddied.

## 5. The Grounds for Skepticism

Few people in North America today can be unaware of the fact that there has been much controversy about the benefits of breast screening. Unquestionably, screening advocates are dominant. However screening skeptics deserve to be heard.

Consider two trials, Trial A and Trial B.

Trial A has informed consent and individual randomization. Trial B has no informed consent and uses cluster randomization.

Trial A maintains consistent numbers of participants and deaths over years of follow-up. Trial B does not [19].

Trial A has 100% compliance at first screen; not so for Trial B.

Trial A uses two-view mammography, Trial B single-view mammography.

Trial A screens every 12 months. Trial B screens every 24–33 months.

Trial A has an external audit of mammography based on stratified sampling. Trial B does not.

Trial A has a higher cancer detection rate with smaller tumor size at first screen than Trial B [22].

Trial A has external pathology reviews to confirm all biopsies performed. Trial B does not.

Trial A has an external death review panel to determine cause of death in all cases of deaths in participants known to have breast cancer during the trial or suspected of having breast cancer after linkage with a national data base. Not so for Trial B.

Rationally, one would expect that Trial A would be deemed superior to Trial B, but it is Trial B that has recently been described as flawless and meticulously conducted! Trial A is the CNBSS and Trial B is the Two-County trial: the two trials most prominently involved in the screening controversy. The CNBSS showed a null effect of screening and the Two-County trial even though it used only single-view mammography and a frequency of 24–33 months—showed the largest benefit of any trial.

Given the intense criticism directed at the CNBSS, it is puzzling that for decades the screening advocates unquestioningly accepted results from the Two County trial. Rational discourse about screening might have considered the disadvantages of cluster randomization, the lack of informed consent and the absence of demographic data other than age at entry for all participants in the Two-County trial. It did not happen. Nor did screening advocates question the inconsistent numbers in the Two-County trial, not only of participants, but of breast cancer deaths. For more than two decades there was little comment about flawed outcome analysis (determination of breast cancer deaths) in the Two-County trial. Only in 2009, did the Two-County trialists finally address (not entirely convincingly) the number problems in the Journal of Medical Screening, reconciling numbers and explaining why differences were observed [23].

The situation was very different in the CNBSS. Its strengths included the advantages of individual randomization; detailed demographic information from controls on entry; annual follow-up of controls; consistent numbers of participants, breast cancers and breast cancer deaths; and a meticulous and external outcome analysis. A weighted random sample of mammograms from every center was regularly reviewed by a reference radiologist. All breast biopsies and all breast cancer diagnoses were reviewed by panels of external pathologists. Panels of external oncologists reviewed all medical records for any death where there was any suspicion of breast cancer. In each of these reviews, the examiners were blind to screening status.

## 6. Was the Canadian Study Really Flawed?

### 6.1. Randomization

A prominent U.S. radiologist asserted that women aged 40–49 with advanced cancers were “placed” in the screened group [24]. That would be scientific fraud. Due to the need for informed consent from the controls, the study design required that all women visited a screening center before randomization occurred. To tell 50% of study participants aged 40–49 on arrival at the screening centers that they had been randomized to receive nothing was not considered fair or reasonable. Women had to come to the centers, usually requiring considerable travel time, often requiring time off work, and in some cases necessitating baby-sitting. Had they come, signed informed consent, been randomized and then told to go home without receiving any benefit, recruitment might well have been impaired. So the decision was made that all women would receive a minimum of a CBE and instruction in breast self-examination. Randomization was performed by the center coordinators after nurse examiners had clinically examined the participants. Center coordinators were blind to the results of the breast examination.

What in fact was the situation *vis-a-vis* randomization?

Most tellingly there was no incentive for screening personnel to subvert randomization. The CNBSS protocol required that anyone with an abnormal finding on CBE had to be referred to the study surgeon who would order a diagnostic mammogram when clinically indicated. Symptomatic women require diagnostic mammography, not screening mammography. It was not necessary to “place” as claimed [24] clinically positive participants in the mammography arm of the study in order for them to get a mammogram.

In the CNBSS there were more than 50 variables (demographic and risk factors) which were virtually identically distributed across control and study groups, clear evidence of successful randomization [25,26]. In contrast, the only variable available for the Two-County trial to assess the success of randomization was age at entry, and this was not equally distributed across the two groups.

In the age group 40–49, there were more women who were clinically positive in the control group (3,674) than in the mammography group (3,569). This does not support the claim that clinically positive women were preferentially allocated to mammography [24]. If randomization had been subverted, there should be an excess of clinical positives in the mammography arm, not 105 fewer [25]. If it is argued that subversion shifted only the few women destined to die of breast cancer to the mammography arm, the implausible conclusion has to be that CBE has superior prognostic and diagnostic attributes compared to mammography.

Responding to relentless attacks, CNBSS investigators allowed a forensic audit of the randomization sheets; the audit found no evidence of subverted randomization [27].

Proposing that 15 dedicated coordinators in the 15 Canadian centers violated the study protocol is truly calumny. But accusations continue in 2010: “That the CNBSS violated fundamental rules for randomized controlled trials is indisputable.” [28]. It is disputable. And incorrect [29,30].

### 6.2. Mammography

Regarding CNBSS mammography, it was also claimed in 2009 “The CNBSS mammography was indefensibly poor” [24]. In fact, CNBSS mammography achieved outstanding results. For ages 40 to 49, cancer detection rates at the first screen were 2.53 in Canada *versus* only 2.09/1,000 in The Two-County trial, while for women aged 50 to 59 they were 5.48 *versus* only 4.67/1,000 [25,26,31].

If CNBSS mammography was so flawed, how could CNBSS cancer detection rates exceed those of the Two-County trial? And how come the tumors detected in Canada were smaller than those detected mammographically in Sweden [22]. Interestingly, the CNBSS is the only screening study that published results from internal and external audits of mammography [32].

### 6.3. Distortion of CNBSS Results

Another U.S. radiologist reported in 1992 that cancer detection had been delayed for two to five years in almost 20% of screen-detected breast cancers in the CNBSS [33]. A two-year delay in diagnosis is possible; however, four-and five-year delays are unbelievable! He claimed that 28 cancers could have been found two years earlier, 33 cancers three years earlier, 27 cancers four years earlier and 12 cancers five years earlier. He claimed he was citing CNBSS results. In fact, the article he cited had reported that on retrospective review, there were 28 cancers at the second screening visit that were mammographically detectable one year earlier at the first screen, 33 cancers at the third screening visit that were detectable one year earlier, 27 at the fourth and 12 at the fifth screen [32]. Similar data have been released by no other screening trialists.

This distortion of the CNBSS published results was just too delicious to be curtailed, and so the information continued to be repeated [34,35]. Interestingly, the same critic reported in another paper that a false negative rate of 54% (when radiologists reviewed prior mammograms) was illustrative of a well known phenomenon, namely that even expert reviewers can fail to observe abnormalities [36]. False negatives do occur, but in the CNBSS for 44,718 women age 40–59, the false negative rate was only 25%, the sensitivity was 75% and the specificity was 94% [37]. Thus, “flawed” mammography in the CNBSS yielded a false negative rate half that condoned clinically.

### 6.4. Disseminating Nonsense

Assertions were made on the Internet in 2000 that according to a Bedford, Virginia radiologist the “Canadian Study did not even use mammography equipment – they were using regular X-Rays!” This was disseminated in spite of two articles in peer-reviewed radiology journals that described in detail the mammography units used in the CNBSS centers [37,38]. More nonsense: Dr. Stephen Edge, a U.S. surgeon, was told at a U.S. medical meeting in 2000 that Canadian hospitals were so poor that they had to send their breast cancer patients to get free mammograms [30]. Even a prestigious journal such as Science reported that the nurse-examiners randomized participants after doing their clinical examination. The source of this misinformation was a radiologist [39].

## 7. Attacking the United States Preventive Services Task Force

Screening advocates have not restricted their criticisms to the CNBSS. A firestorm was unleashed by the November 2009 release of the United States Preventive Services Task Force (USPSTF) Guidelines for Breast Screening [40]. According to the *British Medical Journal* “The recommendations were widely and loudly denounced by radiologists, breast cancer survivors, media doctors, gynecologists and politicians. Medical experts called the task force ‘idiots’ and conservatives lined up to denounce the report as an Obama administration plot” [41]. In contrast, a recent *New England Journal of Medicine* article emphasized the importance of objective agencies such as the USPSTF in evaluating health care initiatives. The conclusion was that “we can work to prevent vested interests from having the loudest voices in health care.” [42]. These voices are loud as illustrated below.

Responding to the 2009 USPSTF guidelines the American College of Radiology declared that “two decades of decline in breast cancer mortality could be reversed and countless American women may die needlessly from breast cancer each year” [43]. This claim ignores the evidence that breast cancer mortality has declined in many jurisdictions due to improved therapy—not only in the absence of screening but also in women too young to be eligible for screening [44-47]. The College additionally claimed that the USPSTF guidelines were “flawed, shocking and unconscionable”. Interestingly, The American College of Radiology has received donations of at least $1 million each from GE Healthcare and Siemens AG. Both companies make mammography equipment and MRI scanners. The lobbying group that led the charge in Washington against the new USPSTF guidelines includes GE, Siemens and the American College of Radiology [43]. Currently ‘vested interests’ seem to be unrecognized by or of concern to the public.

Criticisms directed at the USPSTF by a single screening advocate are revealing.

He claimed that the USPSTF was telling women to wait until their breast cancers were so large they could no longer be ignored and asserted that the USPSTF was incompetent [43]. Elsewhere he said the USPSTF “does not think it is worth saving women in the 40s; it thinks that women should be allowed to die from their breast cancers.” [48]. Furthermore, he stated that the guidelines are “not supported by scientific evidence and should be rescinded” [49].

Of those who disagree with him he charged that “They distort data, rely on weak science, but refuse to defend when challenged” and that “Many European countries as well as Canada do not support or at any rate do not encourage screening before the age of 50 ***and have lied to their populations***”[50]. And when he disagreed with a published article, his response was that the peer-reviewers were incompetent and the article should not have been published [51]. Clearly rhetoric runs amok when screening benefits are challenged.

## 8. The Downsides of Mammography Screening

Many are familiar with at least some of the downsides:

Anxiety either before a screening appointment or after a positive call has been made on a mammogram.

False-positives, which can affect up to 30% of women who are screened multiple times in their 40s, leading to unnecessary biopsies [51].

False-negatives when a cancer diagnosis is delayed and the woman is falsely reassured. Mammography is never 100% accurate [36].

However there are other downsides that have been more recently recognized.

Over-diagnosis, represented by ductal carcinoma *in situ*, which seems to constitute 25% and more of all screen-detected cancers leading to unnecessary treatment for breast cancer [21,51]. There is also over-diagnosis of invasive cancers [51,52].

An anomalous increase in mastectomies in screened women [19,52].

The mortality paradox where more screened women age 40–49 die of breast cancer in the first few years after screening is initiated, compared to controls [2].

### 8.1. Over-Diagnosis

Is over-diagnosis real or imagined? Estimates of its magnitude range from trivial to substantial. When the trials were being planned, it was predicted that screening would initially result in an increased incidence of early cancer and that later there would be a corresponding decline in invasive cancer. In fact, U.S. SEER data reveal that the expected correlation between an initial increase in early cancers and later decline in invasive cancers did not occur in the period 1983–2005. The early increase occurred but there was no subsequent decline in advanced cancers [51]. Similar observations have been made in Europe [19,52].

### 8.2. Excess Mastectomies

A review of eight screening trials concluded that screened women had a 20% significantly elevated risk of having a mastectomy compared to control women [19]. In light of the expectation that early detection would result in less aggressive surgery, this is unexpected. Others have made similar observations [53].

### 8.3. Mortality Paradox

In my role as Deputy-Director of the Canadian National Breast Screening Study, I assembled incoming data on new breast cancer cases and any deaths in the study participants. Recruitment had begun in January 1980 but already by 1982 a trend for breast cancer deaths in women age 40 to 49 years on entry had become evident and would persist for some time. To my then amazement, the death count for women who had been diagnosed with breast cancer was always higher in the group allocated to mammography screening than in the control group.

Because the observed excess defied all our expectations, I labeled it the mortality paradox. By the mid 80s, I was discussing it at meetings because exactly the same paradox had been observed early in the New York Health Insurance Plan Study and the compromised Edinburgh study [54]. More importantly, it had been clearly displayed as 26% excess mortality in screened women in the 1985 *Lancet* report on the Two County trial [31]. In every study the paradox was restricted to women in their 40s. However, I quickly learned it was not politically correct to mention the paradox. After I described it at a professional meeting, another speaker (a radiologist) jumped to her feet and bluntly accused me of being unethical and irrational for mentioning the phenomenon in public. Surely suppressing scientific observations is what is unethical. Believing then that a biological mechanism must exist, I speculated that timing of surgery relative to the menstrual cycle, or some kind of push-pull mechanism involving primary tumor and metastases, or an estrogenic *milieu*, might explain what was happening. However, none of my colleagues were interested in pursuing the matter. Indeed my interest seemed to discomfit them.

By 1997, Cox reported an overview of all trials to date at the NIH Consensus Conference. He showed that excess breast cancer mortality persisted in screened women up until 11 years post entry [2]. But this paper is rarely cited. And it would not be until 2002 that I was able to publish anything about the paradox, and then only under the title ‘Mammography screening: Are women really giving informed consent?’ [55].

Even in 2010 when there is increasing skepticism about the efficacy of breast screening, the main argument focuses on the rather small benefit in terms of breast cancer deaths avoided relative to the much larger likelihood of over-diagnosis and unnecessary treatment. [40]. Never a word about the mortality paradox. Yet there is a long history of research relating to the mortality paradox. As long ago as the mid 19th century, it was observed that surgery appeared to accelerate growth of breast cancer and its fatal termination [56]. In 1983, Bernard Fisher *et al.* reported that following primary tumor removal, kinetic changes in residual tumor tissue (distant metastases) were associated with more rapid growth of metastases [57]. In 1989, Fisher and colleagues concluded: “The findings presented refute the premise that removal of a primary tumor is a local phenomenon with no other biological consequences. They indicate that, following primary tumor removal, metastatic behavior may be affected by an interplay of growth factor(s) which can influence the outcome of a host to its tumor.” [58]. Also in 1989, Judah Folkman listed evidence supporting the hypothesis that tumor growth is dependent on angiogenesis [56,59,60]. He proposed a possible biological mechanism, namely that surgery to remove the primary tumor triggered growth factors to enhance wound healing that also enhanced angioneogenesis. It, in turn, would stimulate the growth of dormant micro-metastases. In recent years the paradox has been thoroughly investigated by Hrushesky, Retsky, Demicheli and colleagues. Their work has been illuminating.

Fisher and Retsky *et al.* [56,58] have convincingly proposed a paradigm shift with respect to how cancer progresses, even if it is being largely ignored. The shift may continue to be ignored until an innovative and very effective therapeutic regime can be tied to it. To my knowledge, a paradigm-related therapy, namely the use of anti-angioneogenesis agents to clinically suppress cancer, has not been hugely successful. For some this may render the paradigm null and void. However, at least at the conceptual level, treating cancer by starving cancer cells specifically is much more appealing than killing cells in general. How can the (not so) new paradigm be made more compelling? Should it be used as an argument against breast screening in women aged 40–49? Where are the curiosity-driven researchers? What are the clinical applications? What ideas have yet to be explored? Why the silence from patient advocates? Or, in short, is the new paradigm at a dead-end?

## 9. Conclusions

There is much to be learned from the breast screening controversy. It boils down to always being ready to question your own beliefs, to look for conflict of interest in those with the loudest voices, to be curious about the unexpected and to do more good than harm.
